# Efficacy and safety of totally laparoscopic gastrectomy with uncut Roux-en-Y for gastric cancer: a dual-center retrospective study

**DOI:** 10.1186/s12957-023-03154-y

**Published:** 2023-09-13

**Authors:** Yizhen Chen, Yuanyuan Zheng, Song Tan, Yifan Chen, Tao Zheng, Shaolin Liu, Yulong Mi, Shentao Lin, Changshun Yang, Jian Jiang, Weihua Li

**Affiliations:** 1 Shengli Clinical Medical College of Fujian Medical University, Fuzhou, 350001 China; 2 Department of Surgical Oncology, Fujian Provincial Hospital, Fuzhou, 350013 China; 3 Department of VIP Clinic, Fujian Provincial Hospital, Fuzhou, 350013 China; 4 Department of Gastrointestinal Surgery, The First Affiliated Hospital of Fujian Medical University, Fuzhou, 350004 China; 5 The School of Public Health, Fujian Medical University, Fuzhou, 350001 China

**Keywords:** Gastric cancer, Totally laparoscopic gastrectomy, Uncut Roux-en-Y, Digestive tract reconstruction

## Abstract

**Background:**

Uncut Roux-en-Y (URY) effectively alleviates the prevalent complexities connected with RY, such as Roux-en-Y stasis syndrome (RSS). Nevertheless, for gastric cancer (GC) patients, it is still controversial whether URY has an impact on long-term prognosis and whether it has fewer afferent loop recanalization. Therefore, compare whether URY and RY have differences in prognosis and long-term complications of GC patients undergoing totally laparoscopic gastrectomy (TLG).

**Methods:**

We analyzed the data of patients who underwent TLG combined with digestive tract reconstruction from dual-center between 2016 and 2022. Only patients undergoing URY and RY were selected for analysis. Relapse-free survival (RFS) and overall survival (OS) were estimated. Bias between the groups was reduced by propensity score matching (PSM). The Cox proportional hazard regression model was used to further analyze the influence of URY on prognosis.

**Results:**

Two hundred forty two GC patients were enrolled. The URY had significantly shorter operation time, liquid food intake time, and in-hospital stays than the RY (*P* < 0.001). The URY had fewer long-term and short-term postoperative complications than the RY, especially with regard to RSS, reflux esophagitis, and reflux gastritis. The 3-year and 5-year OS of the URY group and the RY group before PSM: 87.5% vs. 65.6% (*P* < 0.001) and 81.4% vs. 61.7% (*P* = 0.001). PSM and Cox multivariate analysis confirmed that compared to RY, URY can improve the short-term and long-term prognosis of GC patients.

**Conclusion:**

TLG combined with URY for GC, especially for advanced, older, and poorly differentiated patients, may promote postoperative recovery and improve long-term prognosis.

## Background

Radical surgery is currently recommended for patients with initially operable gastric cancer (GC) [1–4]. With the innovation of laparoscopic instruments and the widespread application of minimally invasive technologies in GC [5, 6], laparoscopy-assisted gastrectomy (LAG) has transitioned to totally laparoscopic gastrectomy (TLG) [7–9].

For digestive tract surgery, different anastomotic methods may affect postoperative complications and short-term prognosis. As a traditional anastomotic method, Roux-en-Y (RY) is widely applied in laparoscopic distal gastrectomy (LDG) or laparoscopic total gastrectomy (LTG) [10, 11]. However, RY is often accompanied by Roux stasis syndrome (RSS) and so on [12, 13]. These complications seriously affected postoperative recovery and quality of life (QoL) [14]. Currently, uncut Roux-en-Y (URY) is most often applied to LDG [15], and relatively little has been reported in LTG [16]. URY has fewer postoperative complications than RY [17, 18]. However, whether or not URY will have afferent loop recanalization [19] and a better long-term prognosis remains controversial.

Existing studies on URY mainly focus on laparoscopic-assisted operations. However, there are few studies on TLG combined with URY. Whether the complications after digestive tract surgery affect the long-term survival of GC patients is a hot topic at present. Following the striking results of previous studies, this study was also surprised by the impact of the surgical technique on prognosis [20]. Therefore, we added data from another center to confirm whether URY is also applicable to totally LDG (TLDG). This study reviewed the dual-center data (including totally LTG (TLTG) and TLDG) to compare whether URY and RY differ in long-term survival under TLG.

## Materials and methods

### Grouping and study population

We conducted a dual-center retrospective comparative study. Clinical information of patients (January 2016 to January 2022) who underwent TLDG or TLTG combined with digestive tract reconstruction at Fujian Provincial Hospital and the First Affiliated Hospital of Fujian Medical University were analyzed. Screen GC patients according to the following requirements: (1) diagnosis as gastric adenocarcinoma by preoperative endoscopy and pathology; (2) the TNM stage (I to III); (3) receiving TLDG or TLTG combined with digestive tract reconstruction; (4) clinical information is complete (including preoperative and postoperative endoscopy, computed tomography (CT), pathology); (5) digestive tract reconstruction limited to URY or RY. Exclusion criteria: (1) history of other malignant tumors; (2) conversion to laparotomy or small incision-assisted anastomosis; (3) non-surgical radical therapy was performed before surgery, such as radiotherapy, chemotherapy, or endoscopic resection; (4) emergency operation; (5) patients lost to follow-up. Eligible patients were automatically grouped according to the chosen anastomotic procedure at the time of surgery (either RY or URY), for the purpose of comparison. The anastomosis method was decided by the surgeon and the patient through consultation before operation. All GC patients received written informed consent. This study was approved by the Medical Ethics Committee of the two centers.

### Surgical procedure

TLG was performed on all GC patients by the same surgical team at both medical centers. The surgeons of the two centers have more than 600 cases of experience with TLG. The surgical approach is created using the “five-port method.” Abdominal exploration was performed initially to rule out obvious metastases. All patients underwent standard TLG [21]. The steps of digestive tract reconstruction of TLTG have been described in a previous study [20]. The steps of digestive tract reconstruction of TLDG were as follows:

#### URY anastomosis

An incision was made on the jejunum, which was about 25 ~ 30 cm from the Treitz ligament. And the linear cutting closure was placed into the residual stomach and jejunum incision respectively. The joint opening between the residual stomach and jejunum was closed to form the food outflow pathway. An incision was made at the input loop of jejunum about 10 cm to the anastomosis. Similarly, an incision was made at the output loop jejunum about 35 cm to the gastrointestinal anastomosis. Then, we performed the jejunal Braun anastomosis and closed the input loop of jejunum, which was 2 ~ 3 cm distal to the gastrointestinal anastomosis, using 6-row nail uncut linear cutting closure [20]. Finally, the closure was reinforced with sutures.

#### RY anastomosis

An incision was made on the jejunum, which was about 25–30 cm from the Treitz ligament. And linear cutting closure was placed into the residual stomach and jejunum incision respectively. The joint opening between the residual stomach and jejunum was closed to form the food outflow pathway. The input loop of the jejunum, about 2–3 cm from the gastrointestinal anastomosis, was cut off by the linear cutting closure. An incision was made on the proximal jejunal stump and the output loop jejunum 35–40 cm from the gastrointestinal anastomosis. Then, the jejunal Braun anastomosis was performed with a linear cut closure.

### Definitions

The overall survival (OS) was the endpoint of this study. The long-term complications associated with digestive tract reconstruction and afferent loop recanalization were the secondary endpoints. Relapse-free survival (RFS): from TLG to the last follow-up or recurrence [20]. OS: from TLG to the last follow-up or death [20]. The short-term complications were defined as those that occur within 30 days of operation (Clavien-Dindo) [22]. Long-term complications were those that occurred during follow-up, including reflux gastritis or esophagitis, anastomotic stenosis, dumping syndrome, and RSS. RSS is defined as follows: (1) RY or URY was used for digestive tract reconstruction; (2) there were still vomiting, nausea, abdominal distension, and other gastrointestinal symptoms for more than 3 months after gastrectomy; (3) food residue in Roux loop endoscopic and imaging examination. Exclusions were made for mechanical intestinal obstruction, anastomotic stenosis, ulcers, and tumor recurrence as potential causes. [20, 23, 24]. All patients underwent endoscopy and gastrointestinal radiography every 3 to 6 months within the first 2 years after surgery. The frequency of examination can be increased according to the symptoms of patients. The postoperative feeding regimen was the same in both centers. Specific adjustments should be made according to postoperative intestinal function recovery.

### Follow-up

Follow-up protocols were conducted as followed: If GC patients did not progress within the first 2 years, follow-up was performed at 6-month intervals; If GC patients did not progress within the first 5 years, follow-up was performed at 1-year intervals. Endoscopy, gastrointestinal radiography, and CT are required to confirm complications and disease progression. The study was followed up until January 1, 2023. Follow-up included survival status, long-term complications, and so on. For the diagnosis of afferent loop recanalization, patients need to be followed up for 1–2 years. For patients with suspected afferent loop recanalization, after combining endoscopy and CT (excluding tumors, mechanical intestinal obstruction, etc.), this study will further use upper gastrointestinal radiography to confirm the diagnosis. If it is found that the contrast medium reaches the other side of the digestive tract through the uncut position of the operation, the diagnosis is established.

### Statistical analysis

According to the data types, use the continuity correction, or Pearson’s chi-squared, or Student’s *t*-test to respectively compare perioperative and basic information. For prognosis, estimate the OS or the RFS by Kaplan–Meier (KM) method and compare the two groups. The Cox proportional hazards model was used to explore independent factors affecting OS or RFS [25]. Firstly, perform univariate analysis. Then, the relevant factors (*P* < 0.1) were included in the multivariate analysis. All results with *P* < 0.05 were considered significant [20].

To improve the reliability of the study, propensity score matching (PSM) was used to eliminate the effect of baseline imbalance [20]. Multiple logistic regression was used to measure propensity scores for each patient [20, 26]. Operative method, TNM stage, and differentiation degree were included as matching variables. Use a 0.02-width caliper to perform one-to-one nearest-neighbor matching. The SPSS software was performed for statistical analysis (version 25, SPSS Inc., Chicago, IL, USA).

## Results

### Clinicopathological characteristics

**Fig. 1:**
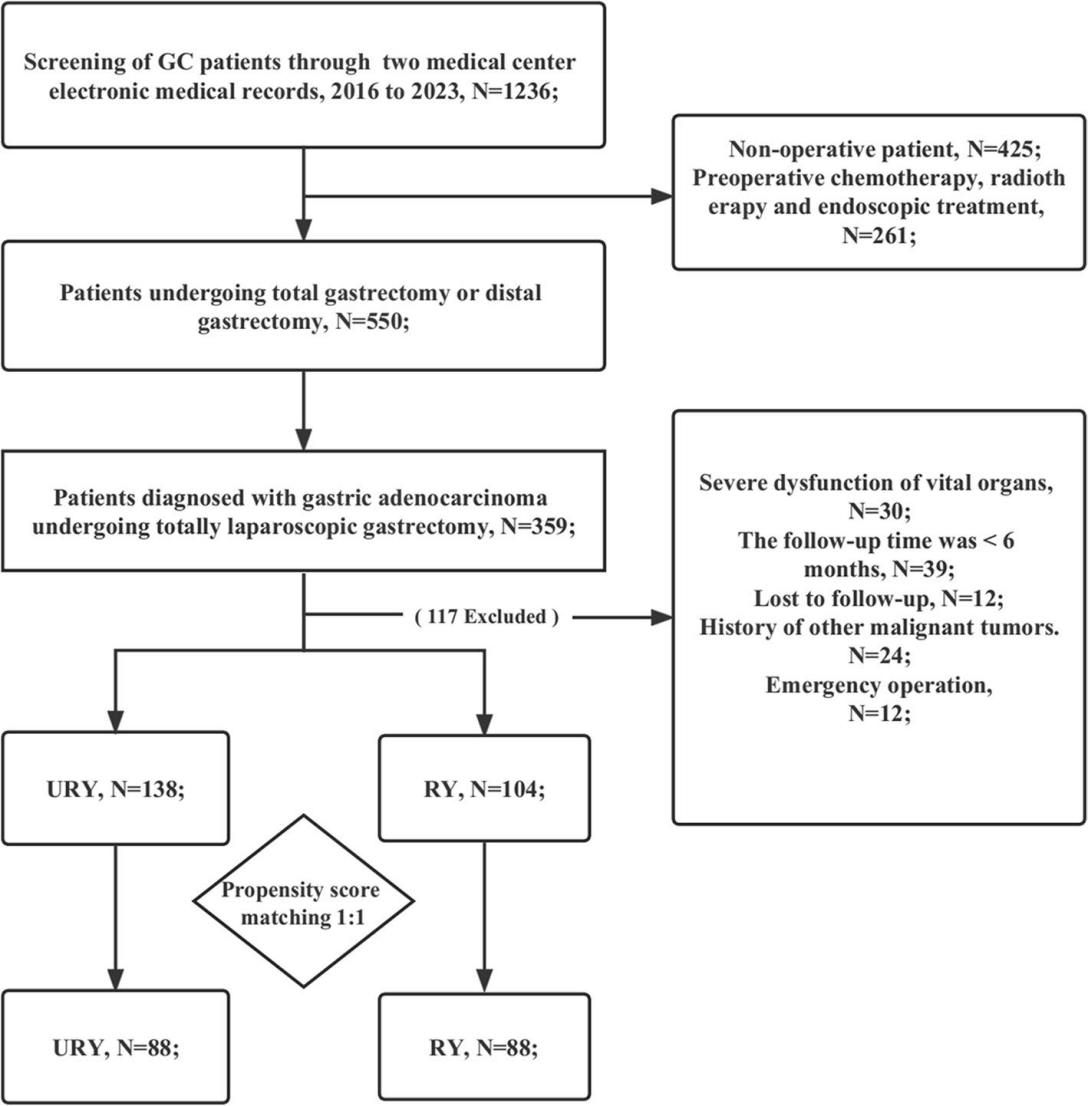
Flow chart of GC patients receiving TLG combined with RY or URY

A total of 242 patients from the two medical centers were enrolled (January 2016 to January 2022) (Fig. 1). The above selection process was conducted independently by two authors (Yizhen Chen and Song Tan). In the event of any disagreement, the third author (Weihua Li) would intervene to solve the problem. There were 178 cases at Fujian Provincial Hospital and 64 at the other hospital. After grouping based on anastomosis, there were 138 cases in the URY group and 104 in the RY group (Table 1). In the entire cohort, male patients accounted for the majority (*N* = 171, 70.37%). There are differences in the initial baseline in surgical methods and TNM staging. None of the patients received radiotherapy and chemotherapy before gastrectomy. For each year from 2016 to 2022, a similar proportion of the two groups was enrolled, which ensured that surgical proficiency would not affect the final outcome.

**Table 1:** Baseline characteristics of the including GC patients

	Before PSM			After PSM		
Variables	URY	RY	*P*^†^	URY	RY	*P*^†^
*N*	138	104		88	88	
Age (years)						
≤ 60/ > 60	52/86	45/59	0.380	34/54	39/49	0.444
Gender						
Female/male	44/94	27/77	0.316	28/60	23/65	0.406
ASA						
I–II/III	103/35	84/20	0.260	67/21	73/15	0.262
Operative method						
TLTG/TLDG	65/73	35/69	0.035	32/56	35/53	0.641
T stage						
T1–T2/T3–T4	72/66	37/67	0.010	30/58	32/56	0.752
LN metastasis						
N0/N +	81/57	44/60	0.012	39/49	40/48	0.880
TNM						
I + II/III	98/40	51/53	0.001	48/40	46/42	0.762
Differentiation degree						
High + medium/low	70/68	64/40	0.094	47/41	48/40	0.880

^†^Pearson’s *x*^2^ test was used to analyze the basic characteristics

*GC* gastric cancer, *PSM* propensity score matching, *ASA* American Society of Anesthesiologists, *TLTG* totally laparoscopic total gastrectomy, *TLDG* totally laparoscopic distal gastrectomy, *LN*,Lymph node

### Perioperative information

The perioperative conditions of GC treated by TLG are shown in Table 2. Two groups completed TLG (including TLTG and TLDG). None of the GC patients were converted to laparotomy. The URY group had a significantly shorter operation time, shorter length of hospital stays (LOS), and shorter anastomosis time than the RY (all *P* < 0.001). And the URY also had an advantage over the RY group in terms of intraoperative blood loss (mean 95.07 mL vs. 176.56 mL; *P* < 0.001). As for the 30-day postoperative mortality, there were zero cases in the URY and two cases in the RY (one case was due to a postoperative complication, and the other one had an unknown cause). The URY group had a higher proportion of postoperative chemotherapy (44.20% vs. 32.69%, *P* = 0.069). As for the incidence of short-term postoperative complications, RY was higher than URY (30.77% vs. 11.59%, *P* < 0.001). Pneumonia was the most postoperative short-term complication. Moreover, the number of serious postoperative complications in the RY group was more than twice as high as those in the URY group.

**Table 2:** Perioperative outcomes of GC patients

Variables	URY	RY	*P*^†^
*N*	138	104	
Operative time (min)	204.83 ± 49.85	229.08 ± 51.44	< 0.001
Intraoperative anastomosis time (min)	35.37 ± 9.38	45.63 ± 8.13	< 0.001
Intraoperative blood loss (ml)	95.07 ± 49.47	176.56 ± 165.24	< 0.001
LOS (days)	8.29 ± 2.55	10.33 ± 4.45	< 0.001
Intake time of liquid food (days)	2.63 ± 0.87	3.98 ± 1.97	< 0.001
Postoperative chemotherapy; *N* (%)	61 (44.20)	34 (32.69)	0.069
Postoperative mortality in 30 days; *N* (%)	0 (0.00)	2 (1.92)	0.358
Overall short-term postoperative complications; *N* (%)^a^	16 (11.59)	32 (30.77)	< 0.001
Pneumonia; *N* (%)	4 (2.90)	7 (6.73)	0.269
Fever; *N* (%)	2 (1.45)	6 (5.77)	0.134
Pleural effusion; *N* (%)	2 (1.45)	6 (5.77)	0.134
Gastric paralysis; *N* (%)	2 (1.45)	4 (3.85)	0.442
Other; *N* (%)	6 (4.35)	9 (8.65)	0.169
Serious complications (Clavien III-V); *N* (%)	5 (3.62)	10 (9.62)	0.056

*GC* gastric cancer, *LOS* length of hospital stay

^†^Continuity correction, Pearson’s *x*^2^ test, or Student’s *t*-test was used to analyze the basic characteristics

^a^According to the Clavien–Dindo classification

One of the focuses of current research is long-term complications (Table 3). In terms of long-term complication, the RY was 3 times more than the URY (9.42% vs. 13.77%, *P* < 0.001). In terms of the incidence of RSS and reflux esophagitis or reflux gastritis, RY was higher than URY (all *P* < 0.05). No difference was found in other long-term complications. There was no afferent loop recanalization of URY group during follow-up regarding the controversial hot spots.

**Table 3:** Long-term postoperative complications

Variables	URY	RY	*P*^†^
N	138	104	
Overall long-term complications; *N* (%)	19 (13.77)	41 (39.42)	< 0.001
RSS; *N* (%)	9 (6.52)	24 (23.08)	< 0.001
Reflux esophagitis/gastritis; *N* (%)	11 (7.97)	19 (18.27)	0.016
Anastomotic stenosis; *N* (%)	2 (1.45)	6 (5.77)	0.134
Dumping syndrome; *N* (%)	2 (1.45)	6 (5.77)	0.134

*RSS* Roux-Y stasis syndrome

^†^Continuity correction and Pearson’s *x*^2^ test were used to analyze the basic characteristics

### Survival analysis

All GC patients were followed up with strict standards. Both groups underwent R0 resection. Since some initial baselines were slightly different, we used PSM to address these concerns. The URY and RY groups got a one-to-one patient match (Table 1). There was no difference in the baseline of the matched cohort. To confirm whether there were still differences in complications between the two groups after PSM, we conducted another comparison. Table 4 further confirmed that URY had prognostic advantages regarding blood loss, LOS, postoperative short-term, and long-term complications (*P* < 0.05). However, the URY group had a higher proportion of postoperative chemotherapy (56.82% vs. 31.82%, *P* = 0.001).

**Table 4:** Perioperative information and complications of GC patients after PSM

Variables	URY	RY	*P*^†^
N	88	88	
Operative time (min)	207.94 ± 51.32	230.02 ± 53.03	0.006
Intraoperative anastomosis time (min)	37.25 ± 9.56	45.31 ± 7.84	< 0.001
Intraoperative blood loss (ml)	98.41 ± 47.58	172.73 ± 170.43	< 0.001
LOS (days)	8.24 ± 1.82	10.83 ± 4.60	< 0.001
Intake time of liquid food (days)	2.58 ± 0.84	4.07 ± 2.08	< 0.001
Postoperative chemotherapy; *N* (%)	50 (56.82)	28 (31.82)	0.001
Overall short-term postoperative complications; *N* (%)^a^	12 (13.64)	29 (32.95)	0.002
Overall long-term complications; *N* (%)	15 (17.05)	35 (39.77)	0.001
RSS; *N* (%)	9 (10.23)	18 (20.45)	0.060

*GC* gastric cancer, *PSM* propensity score matching, *LOS* length of hospital stay

^†^Pearson’s *x*^2^ test or Student’s *t*-test was used to analyze the basic characteristics

^a^According to the Clavien–Dindo classification

**Fig. 2:**
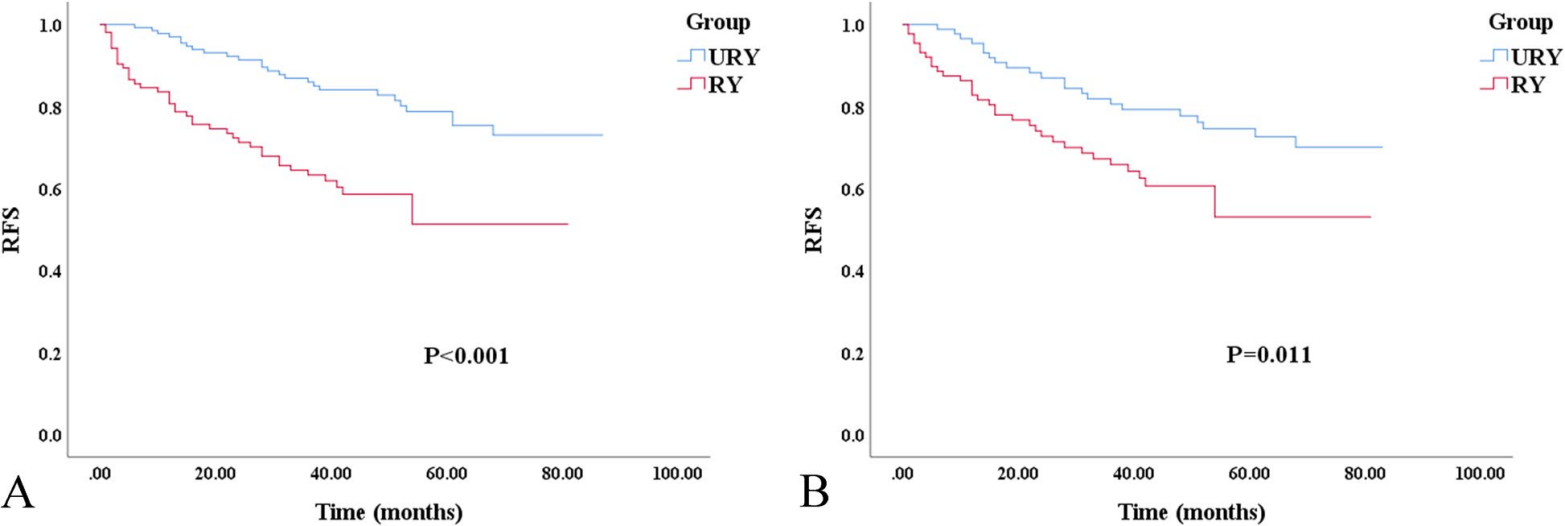
Kaplan–Meier survival curve for RFS of URY group and RY group. **A** Unmatched analyses. **B** Propensity-score-matched analyses

Compared to RY, the URY group showed improvement in RFS (Fig. 2A and B). The 3-year and 5-year RFS of URY and RY were 86.0% vs. 63.3% (*P* < 0.001) and 78.8% vs. 51.2% (*P* < 0.001), respectively. After PSM, the 3-year and 5-year RFS of URY and RY were 80.6% vs. 65.8% (*P* = 0.027) and 74.6% vs. 53.0% (*P* = 0.003), respectively. The poor RFS were independently associated with lymph node metastasis [hazard ratio (HR), 2.961; 95% confidence interval (CI): 1.110–7.901; *P* = 0.030], advanced TNM [HR, 2.896; 95% CI: 1.093–7.669; *P* = 0.032], and the presence of RSS [HR, 5.525; 95%CI: 2.370–12.879; *P* < 0.001] (Table 5).

**Table 5:** Analysis of prognostic factors associated with RFS

Prognostic factor	*n*	Univariate HR (95% CI)	*P*	Multivariate HR (95% CI)	*P*
Group					
URY	138				
RY	104	2.901 (1.742–4.831)	< 0.001	1.621 (0.891–2.948)	0.113
Gender					
Female	71				
Male	171	1.813 (0.988–3.326)	0.055	1.819 (0.964–3.431)	0.065
Age (years)					
≤ 60	97				
> 60	145	1.563 (0.931–2.625)	0.091	1.460 (0.852–2.502)	0.169
ASA					
I–II	187				
III	55	0.628 (0.320–1.231)	0.176		
T-stage					
T1 + T2	109				
T3 + T4	133	7.919 (3.777–16.605)	< 0.001	2.135 (0.803–5.671)	0.128
LN metastasis					
No	125				
Yes	117	5.537 (3.061–10.016)	< 0.001	2.961 (1.110–7.901)	0.030
TNM					
I + II	149				
III	93	8.302 (4.648–14.828)	< 0.001	2.896 (1.093–7.669)	0.032
Differentiation degree					
High + Medium	134				
Low	108	1.334 (0.823–2.163)	0.241		
Overall short-term complications					
No	194				
Yes	48	2.183 (1.298–3.671)	0.003	1.782 (0.993–3.199)	0.053
Overall long-term complications					
No	182				
Yes	60	4.450 (2.740–7.227)	< 0.001	1.974 (0.856–4.557)	0.111
RSS					
No	209				
Yes	33	7.758 (4.715–12.766)	< 0.001	5.525 (2.370–12.879)	< 0.001

*RFS* relapse-free survival, *HR* hazard ratio, *LN* lymph node, *RSS* Roux-Y stasis syndrome

**Fig. 3:**
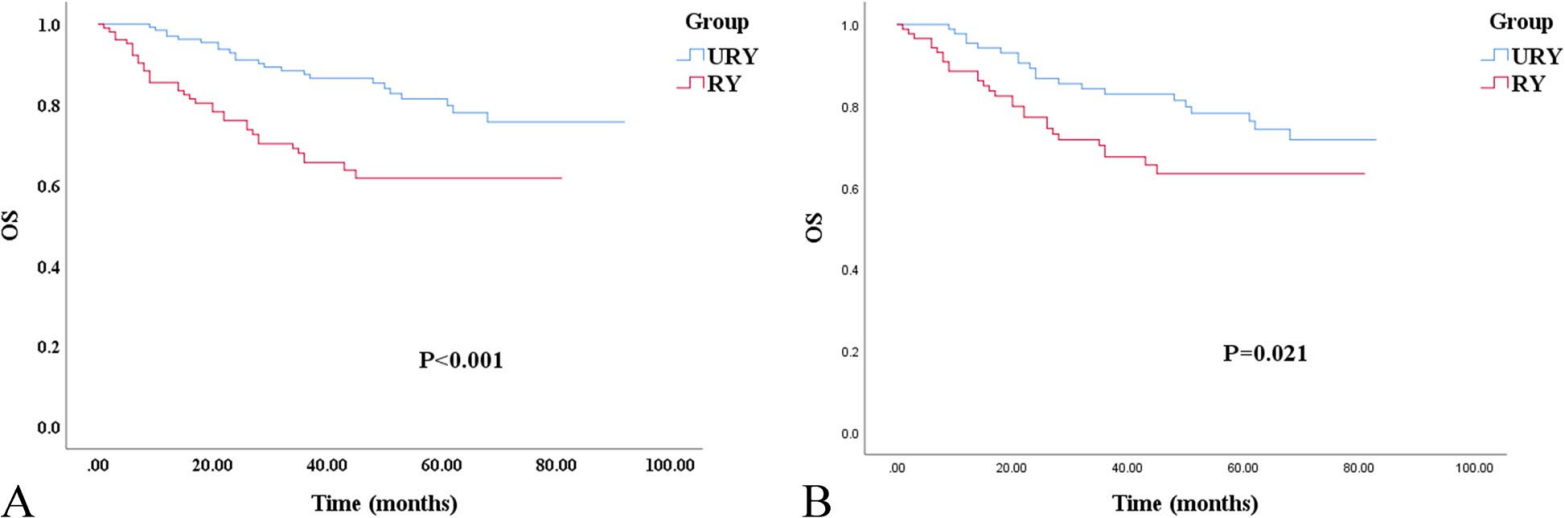
Kaplan–Meier survival curve for OS of the URY group and RY group. **A** Unmatched analyses. **B** Propensity-score-matched analyses

Compared with the RY, the OS of the URY was improved (Fig. 3). The 3-year and 5-year OS of the URY and RY were 87.5% vs. 65.6% (*P* < 0.001) and 81.4% vs. 61.7% (*P* = 0.001), respectively. After PSM, 3-year and 5-year OS of the URY and RY were 82.9% vs. 67.5% (*P* = 0.015), and 78.2% vs. 63.4% (*P* = 0.021), respectively. The poor OS was independently associated with advanced TNM staging (HR, 3.179; 95% CI: 1.014–9.966; *P* = 0.047) and RSS (HR, 3.956; 95% CI: 1.599–9.787; *P* = 0.003) (Table 6). In addition, Cox multivariate analysis showed that anastomosis had no prognostic effect on RFS and OS.

**Table 6:** Analysis of prognostic factors associated with OS

Prognostic factor	*n*	Univariate HR (95% CI)	*P*	Multivariate HR (95% CI)	*P*
Group					
URY	138				
RY	104	2.808 (1.630–4.836)	< 0.001	1.639 (0.892–3.013)	0.111
Gender					
Female	71				
Male	171	1.696 (0.898–3.203)	0.103		
Age (years)					
≤ 60	97				
> 60	145	1.514 (0.875–2.622)	0.138		
ASA					
I–II	187				
III	55	0.666 (0.327–1.355)	0.262		
T-stage					
T1 + T2	109				
T3 + T4	133	14.577 (5.274–40.291)	< 0.001	3.264 (0.983–10.837)	0.053
LN metastasis					
No	125				
Yes	117	8.146 (3.992–16.623)	< 0.001	3.141 (0.961–10.265)	0.058
TNM					
I + II	149				
III	93	11.649 (5.870–23.119)	< 0.001	3.179 (1.014–9.966)	0.047
Differentiation degree					
High + Medium	134				
Low	108	1.255 (0.749–2.100)	0.388		
Overall short-term complications					
No	194				
Yes	48	1.890 (1.071–3.334)	0.028	1.687 (0.891–3.196)	0.109
Overall long-term complications					
No	182				
Yes	60	3.679 (2.197–6.162)	< 0.001	1.956 (0.803–4.764)	0.140
RSS					
No	209				
Yes	33	5.872 (3.441–10.020)	< 0.001	3.956 (1.599–9.787)	0.003

*OS* overall survival, *HR* hazard ratio, *LN* lymph node, *RSS* Roux-Y stasis syndrome

### Subgroup analysis

**Fig. 4:**
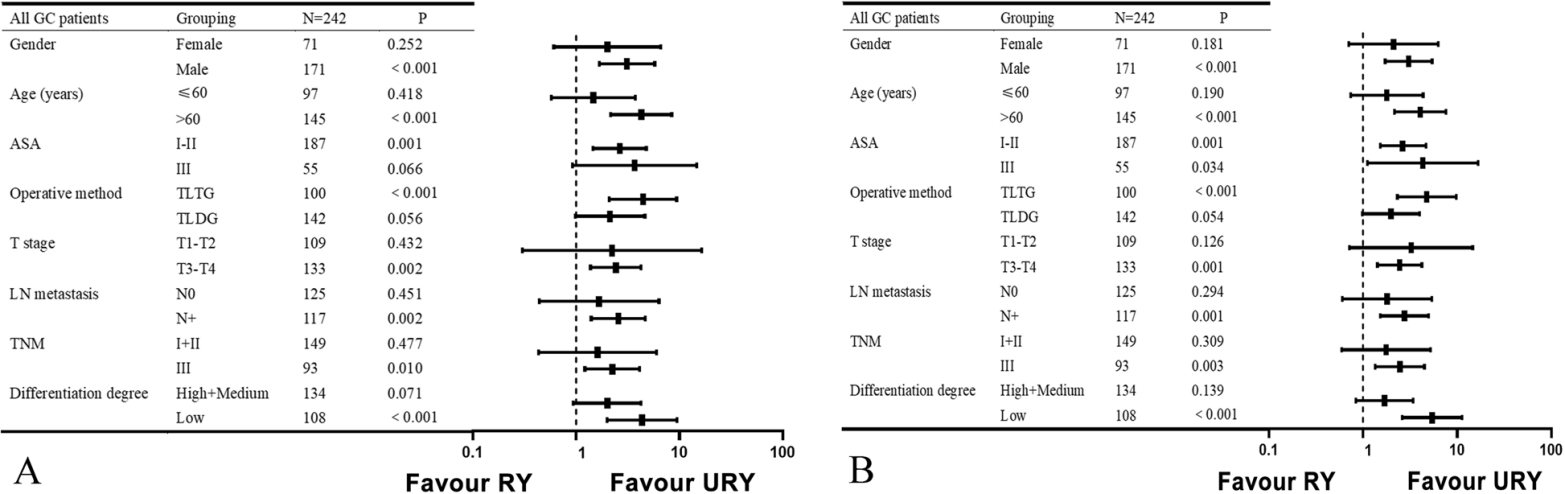
Forest plot evaluating the impact of TLG combined with RY or URY on OS and RFS. **A** Subgroup analysis of OS. **B** Subgroup analysis of RFS. TLTG, totally laparoscopic total gastrectomy; TLDG, totally laparoscopic distal gastrectomy; LN, lymph node

A subgroup analysis was performed to further evaluate whether the two anastomoses still had a prognostic effect on GC patients with varying baseline characteristics. The forest plot reaffirms the previous results (Fig. 4). Notably, the URY still consistently demonstrates superior outcomes in terms of RFS and OS than RY across diverse subgroups. Intriguingly, the advantage of the URY group is particularly pronounced in specific populations, such as those > 60 years old, T stage (III-IV), N stage (N +), TNM stage (III), and exhibiting poor pathological differentiation.

## Discussion

With the advancement of laparoscopic surgery in the field of GC, TLG has been progressively embraced [27, 28]. Totally laparoscopic digestive tract reconstruction demands a high level of technical proficiency from physicians. RY anastomosis was one of the most common surgical procedures of GC in the past few decades [29]. However, RY anastomosis disrupts the normal anatomical structure of the gastrointestinal tract and seriously affects the QoL of patients. In light of its simplicity, URY has gradually been utilized in GC [30]. URY can effectively mitigate RSS arising from digestive tract reconstruction and enhance QoL [20, 29, 31]. However, whether URY entails afferent loop recanalization [32], as well as its potential to ameliorate the long-term prognosis of GC patients, is currently a research focus [20]. Therefore, this study collected patient data from two medical centers to explore whether URY could replace RY.

Through survival analysis, we found that OS and RFS of the URY were better than those of the RY. We implemented PSM to minimize confounding variability between the two groups in order to improve the reliability of the study. PSM effectively simulates randomization of prospective studies [26]. In addition to improving long-term prognosis, URY also has fewer short-term and long-term complications compared to RY. We also found that URY is more suitable for advanced, poor pathological differentiation, and elderly GC patients. This is the first dual-center retrospective study to compare whether TLG combined with URY has a better benefit for GC.

URY has shown advantages in the perioperative period. URY possesses evident benefits in terms of overall operating or anastomosis time. This is attributed to the fact that URY did not sever the jejunum and mesentery vessels [20, 33]. Simplifying surgical procedures can avoid excessive bleeding [16, 17], which is consistent with the findings of this study. In addition, this study found that the URY group had shorter hospital stays. This is because not cutting off the jejunum can not only prevent gastrointestinal dysfunction resulting from retroperistalsis, but also reduce the trauma of small intestine surgery, which greatly alleviates the financial burden on patients and enhances the recovery experience. Short-term postoperative complications determine the speed of postoperative recovery [20]. In this study, the short-term postoperative complications of URY were half as many as those in the RY group. Similarly, research on TLTG combined with URY for GC has confirmed that the postoperative short-term complications of URY were significantly less than those of the RY group [20]. The short-term prognostic advantages of LDG combined with URY for GC were also validated [29]. From the perspective of postoperative recovery, URY is more suitable for GC patients than RY. In addition, it is crucial to consider whether URY has afferent loop recanalization and the impact of anastomosis on long-term prognosis [34].

After gastrectomy, GC patients experience a decline in QoL and nutritional deficiencies [35]. With the advancements in precision medicine, the requirements for GC operation have become increasingly demanding. At present, surgical treatment in the field of GC is more about reducing long-term complications rather than solely improving survival rates. The long-term complications associated with RY anastomosis, especially RSS, were greatly relieved by URY anastomosis [20, 29, 36]. Data from this study showed that the long-term complication of RY was twice that of the URY group (*P* < 0.001). Initially, URY anastomosis was controversial due to the afferent loop recanalization [37]. Two single-center RCTs in 2023 found that afferent loop recanalization occurred in 35.3% and 73.7% of URY patients, respectively [38, 39]. These are inconsistent with these results and other studies [13, 23, 40, 41]. It can be seen that URY is still a controversial hot spot. Combined with the experiences of the two medical centers and relevant literature, this study concluded that the reasons for the recanalization of the afferent loop may be as follows: (1) difficulties in achieving optimal ligature strength with silk thread; (2) inadequate selection of anastomosis location. In this study, 6-row nail uncut linear cutting closure was used (positioned 2 ~ 3 cm away from the gastrointestinal anastomosis); (3) the lack of suture reinforcement at the closure may lead to the recanalization of the afferent loop. During the follow-up period of 6 to 90 months, no recanalization of the afferent loop was observed. URY is an economical and effective option for achieving long-term QoL.

This study yielded unexpected findings, as the URY group exhibited superior RFS and OS compared to the RY group. Previous studies have not discovered that LTG or LDG combined with URY can enhance OS or RFS [17, 40]. This may be due to the low incidence of RSS in GC patients, or it may be related to the different baseline characteristics of the patients. We conclude that URY improves long-term survival by reducing long-term complications, especially RSS. In certain specific populations, the advantage of URY is more pronounced. Cox regression analysis and subgroup analysis supported our conclusion. A single-center RCT in 2023 did not explore the long-term prognosis of patients [38]. Another RCT study found that the long-term prognosis of URY and RY groups was similar [39]. We found that the patients enrolled in the RCT study were early GC. These patients have minimal surgical difficulty and do not require postoperative chemotherapy. As a result, the survival advantage of these patients derived from different anastomosis modalities may not be significant. Because the subgroup analysis of OS and RFS in this study found that advanced patients were more likely to benefit from URY (Fig. 4). Therefore, the results of this study need to be further verified by RCT with large samples (including early and advanced GC patients). The improved OS and RFS of the URY group can be inferred as follows: ① URY improves the possibility of timely utilization of postoperative adjuvant chemotherapy by promoting postoperative rehabilitation. TNM staging is an important prognostic factor for GC patients [42]. The proportion of advanced GC patients was higher in this study, all of whom require postoperative adjuvant chemotherapy to improve prognosis [43]. A prospective randomized controlled clinical trial published in 2019 showed better tolerance of adjuvant chemotherapy in GC patients treated with laparoscopy [44]. This illustrates the importance of quick recovery. This study also fully demonstrates the benefits of fewer postoperative complications and benefits, which are advantageous for timely use of postoperative adjuvant chemotherapy. This is similar to our results. After PSM, the long-term postoperative complication rate in the RY group was nearly three times that in the URY group, and the postoperative chemotherapy rate in the URY group was nearly double that in the RY group. Long-term complications hinder the application of postoperative chemotherapy, a predicament frequently faced by physicians and patients. This is also supported by the subgroup analysis showing that URY has a prognostic advantage in elder or advanced patients. ② URY can reduce postoperative complications. Complications are variables that affect the OS or RFS [45]. ③ Compared to cutting the jejunum, not cutting the jejunum may be more conducive to maintain the intestinal microbial balance. The effects of digestive tract reconstruction on microenvironmental homeostasis and enteral nutrition are intricate. Alterations to the natural anatomy of the gut can exert significant influences on gut barrier function and immunity [46, 47]. ④ A substantial amount of data have shown that increased intraoperative blood loss correlates with a worsened prognosis [48, 49]. URY can reduce intraoperative bleeding. ⑤ URY may reduce intestinal inflammation. The anastomosis may have effects on intestinal inflammation [50]. Therefore, it is necessary to further explore the role of URY in alleviating intestinal inflammation.

There remain certain constraints within this study. Primarily, there were slight differences in initial baseline characteristics. Cox regression analysis or PSM sought to rectify this issue, but the sample size was subsequently reduced. Secondly, data on postoperative nutritional status of patients was not collected. Said parameter directly affects the QoL of the patients. However, the loss of data is related to the inherent shortcomings of retrospective studies. Finally, retrospective studies have problems pertaining to data loss, such as gene mutations. These shortcomings will prompt further prospective randomized controlled trials.

## Conclusion

TLG combined with URY presents both safety and feasibility. TLG combined with URY is completely able to avoid the controversial hot spot (afferent loop recanalization). Especially for advanced, elderly GC patients with poor pathological differentiation, URY anastomosis is recommended as a primary option for digestive tract reconstruction. URY might enhance long-term prognosis by shortening operating time, facilitating postoperative recovery, and potentially through other mechanisms. Further validation of the URY in TLG requires a large sample prospective clinical trial.

## Data Availability

Data for this study may be requested from the corresponding author where appropriate.

## Abbreviations

GC: Gastric cancer
LAG: Laparoscopy-assisted gastrectomy
TLG: Totally laparoscopic gastrectomy
RY: Roux-en-Y
URY: Uncut Roux-en-Y
LDG: Laparoscopic distal gastrectomy
LTG: Laparoscopic total gastrectomy
RSS: Roux stasis syndrome
QoL: Quality-of-life
OS: Overall survival
RFS: Relapse-free survival
